# Medical Cannabis and Past-Year Cannabis Use Disorder Among Adult Recreational Users in the United States: Results From a Nationally Representative Sample

**DOI:** 10.3389/fpsyt.2022.836908

**Published:** 2022-04-01

**Authors:** Dafna Sara Rubin-Kahana, Ahmed Nabeel Hassan, Marcos Sanches, Bernard Le Foll

**Affiliations:** ^1^Child, Youth, and Family Services, Centre for Addiction and Mental Health, Toronto, ON, Canada; ^2^Department of Psychiatry, Faculty of Medicine, University of Toronto, Toronto, ON, Canada; ^3^Department of Psychiatry, King Abdul-Aziz University, Jeddah, Saudi Arabia; ^4^Campbell Family Mental Health Research Institute, Centre for Addiction and Mental Health (CAMH), Toronto, ON, Canada; ^5^Department of Pharmacology and Toxicology, University of Toronto, Toronto, ON, Canada; ^6^Institute of Medical Science, University of Toronto, Toronto, ON, Canada; ^7^Biostatistical Consulting Service, Krembil Centre for Neuroinformatics, Centre for Addiction and Mental Health, Toronto, ON, Canada; ^8^Department of Family and Community Medicine, University of Toronto, Toronto, ON, Canada

**Keywords:** medical marijuana, cannabis use disorder, DSM-5, concurrent disorders, dependence, addiction, NESARC-III

## Abstract

**Introduction:**

With the increasing number of cannabis users and more jurisdictions allowing medical cannabis, more evidence-based knowledge about the prevalence of cannabis use disorder (CUD) among medical users is greatly needed.

**Objectives:**

To examine and compare the prevalence and severity of CUD and the prevalence of different CUD criteria among two groups: those who combine recreational and medical use vs. those who exclusively use cannabis recreationally. To examine the association between CUD and sociodemographic characteristics, medical conditions, and psychiatric comorbidities between these two groups.

**Methods:**

The National Epidemiological Survey on Alcohol and Related Conditions III data were used, a US nationally representative in-person interview of 36,309 adults aged ≥18 years collected in 2012–2013. The statistical examination included proportion comparison hypothesis testing and linear regressions, all using complex survey design analysis procedures.

**Results:**

Recreational users who used cannabis also for medical purposes had a higher prevalence of CUD in general, as well as mild and moderate CUD than users who used cannabis only for recreational purposes. CUD is more prevalent in recreational, medical users with the following characteristics: young, male, non-white, living in the Midwest, using a greater amount of cannabis, having a concurrent mental disorder, and had CUD before the past year.

**Conclusion:**

Recreational, medical cannabis users have a higher likelihood of having CUD. Although the results should be taken with caution, given the lack of established validity of CUD among medical users, health care professionals who prescribe or recommend the use of cannabis for medical purposes should take this into consideration while evaluating the risks/benefits ratio of cannabis. They need to assess patients' recreational cannabis use, screen for CUD, and educate users about the possible complications caused by cannabis use.

## Introduction

The landscape of medical cannabis (MC) is changing, as more jurisdictions worldwide are allowing cannabis use for medical purposes (1, 2). For example, in the United States, the prevalence of MC use increased from 1.2% in 2013 to 1.6% in 2015 (3). Approximately 10% of the adult population in the United States who use cannabis reported using it medically (4). In states that allow MC, the prevalence is higher and reaches 17% (5). A Canadian survey reported that 28.8% of past-year users used cannabis for therapeutic purposes (6).

Medical users report frequent and daily cannabis use relative to recreational users (RUs) (5, 6). Despite evidence supporting a link between frequent cannabis use and cannabis use disorder (CUD) (7, 8), the finding that states in the United States that legalized MC had an increased rate of CUD after legislation (9), only a handful of studies estimated CUD prevalence among medical users.

Two studies used the National Epidemiologic Survey on Alcohol and Related Conditions—III (NESARC-III) to explore MC use. The first study reported that 41.54% of users who used both recreational and medical cannabis use (RMU) met the Diagnostic and Statistical Manual of Mental Disorders, Fifth Edition (DSM-5) criteria for CUD. In contrast, only 25.25% of RU met the same criteria (10). The second study reported that recreational cannabis users with CUD were more likely to use MC compared with RUs who did not use MC (11). Data from another American national survey [the 2013 National Survey on Drug Use and Health (NSDUH)] demonstrated that 10% of individuals who used recreationally and 11% of those who used medically met criteria for cannabis abuse/dependence (5). A Canadian survey showed that 25.8% of RMU had a positive screen for CUD vs. 10.3% of RU (12).

Only a few studies evaluated the characteristics of medical users with CUD. Among patients with chronic pain, dependency was more common among individuals using cannabis longer and in greater quantities, reporting higher levels of depression and anxiety, and using alcohol or drugs (13). CUD among RMUs was more common among young users (11).

So far, there is no established validity for the diagnosis of CUD in the medicinal context (14), nor the CUD criteria experienced by medical users. In a recent review, Schlag et al. found that the prevalence of 5 of the 11 DSM-5 CUD criteria is unknown based on current literature. In contrast, the data related to the other six criteria are supported by a paucity of studies (14). Given the increased use of MC, the high prevalence of CUD in medical users, and more jurisdictions legalizing MC, it is important to understand the characteristics and the prevalence of CUD among patients treated with MC. To the best of our knowledge, no previous study has examined the association between MC and the severity levels of CUD nor the characteristics of RMU in a nationally representative sample.

Our study has three aims. Our first aim is to examine the 1-year prevalence of CUD severity levels among RMUs compared with RUs. Our second aim is to compare the prevalence of the different DSM-5 criteria for CUD among RMUs and RUs. Our third aim is to investigate the impact of different personal characteristics on CUD risk among RMUs and RUs. Our first hypothesis (H1) is that the use of MC among RUs will be significantly associated with more CUD and a more severe CUD. Given the more frequent cannabis use among RMUs, which can lead to a buildup of tolerance and withdrawal symptoms while not using, our second hypothesis (H2) is that prevalence of CUD criteria in RMUs compared with RUs will be different. Our third and final hypothesis (H3) is that the RMU population will differ in personal characteristics associated with CUD, such as physical morbidity and mental and substance use disorders, and there would not be any significant difference related to sociodemographic factors.

We decided to focus on RMUs, due to two reasons. First, the vast majority of medical users also endorse RU. A recent Canadian survey reported that 80.7% of medical users also use cannabis recreationally (12). In a recent American survey, 85% of medical users also use cannabis recreationally (15). Data from NESARC-III show that 79.15% of medical users also report RU (11). The second reason is the lack of established validity of CUD diagnosis among pure medicinal users.

## Methods

### Sample

The NESARC-III was conducted in the United States in 2012–2013. The participants were 36,309 civilian adults in households and selected group quarters^1^ (16). Respondents were selected through probability sampling, and data were weighted to represent the US population based on the 2012 American Community Survey (17). All respondents were interviewed face-to-face in their homes. The household response rate was 72%, the person-level response rate was 84%, and the overall response rate was 60.1%. NESARC-III methodology is described further elsewhere (16). The NESARC-III was approved by the institutional review board at the National Institutes of Health. The study presented in this article received approval from the Centre for Addiction and Mental Health's Research Ethics Board (099-2019-01).

### Diagnostic Interview

The Alcohol Use Disorder and Associated Disabilities Interview Schedule−5 was used to assess the frequency and amount of drug and alcohol use, DSM-5 substance use disorders, and psychiatric disorders. It is a structured, computer-assisted diagnostic interview designed for lay interviewers (16).

### Cannabis Use and Cannabis Use Disorder

Participants were asked about past-year and before past-year cannabis use. Participants reported either using cannabis recreationally, medically, or both. To assess MC use, responders were asked, “Have you ever been prescribed or used medical marijuana?” (18). All participants who reported RU were assessed for DSM-5 CUD and were asked about the frequency of use and number of joints used on days that cannabis was used.

Consistent with DSM-5, the diagnosis of CUD requires at least 2 of 11 criteria; cases were classified as mild (2 or 3 criteria), moderate (4 or 5 criteria), or severe (6 or more criteria) (19). The test–retest reliability of 12-month and lifetime cannabis use was substantial in a general population sample (20). The test–retest reliabilities of DSM-5 CUD and its dimensional criteria scales (intraclass correlation coefficients = 0.70, 0.71) were fair to substantial in a general population sample (21).

The amount of cannabis used was defined as the number of joints usually smoked in a single day in the past year. The frequency of use was binned as either: every day and nearly every day vs. less frequent cannabis use.

### Medical Conditions

We included in the analysis medical conditions with some empirical support for MC treatment (22) confirmed by a health care professional in the past year. Responders were asked if “During the last 12 months, did a doctor or another health professional tell you that you had…” (18). Medical conditions included were the following: arthritis, nerve problems (combines fibromyalgia, reflex sympathetic dystrophy or complex regional pain syndrome, and any other nerve problem in your legs, arms, or back), insomnia, cancer, and diabetes. Lifetime human immunodeficiency virus or acquired immunodeficiency syndrome were not included due to a limited number of responders, despite having empirical support for MC treatment (22).

Pain was measured with the 12-item Short-Form Health Survey, version 2 (23). Suffering from pain was entered using the question: “During the past 4 weeks, how much did pain interfere with your normal work, including both work outside the home and housework.” Moderately, quite a bit, or extremely were computed as “yes” for pain (10).

### Other Covariates

Sociodemographic covariates included sex (male or female), race/ethnicity (White or others^2^), age (18–29, 30–44, 45 years, or older), marital status (married/living with someone as if married, widowed/divorced/separated, and never married), education (some high school or less, high school graduate, some college, or higher), past-year household income ($0–19,999, $20,000–34,999, $35,000–69,999, $70,000, or greater), urbanicity (urban or rural), and region (northeast, Midwest, south, or west). Psychiatric disorders included: past-year hierarchical^3^ mood disorder (major depressive disorder, dysthymia, and bipolar 1), past-year anxiety disorder (generalized anxiety disorder, social phobia, agoraphobia, specific phobias, and panic disorder), past-year psychotic disorder, and any lifetime personality disorder (borderline, schizotypal, and antisocial).

We decided to control for other substance use disorders (SUDs), as CUD is highly comorbid with other SUDs (24). SUDs included in our analysis are the following: before past-year CUD, past-year alcohol use disorder, past-year tobacco use disorder, past-year other SUDs (cocaine, hallucinogens, opioids, sedatives, inhalants/solvents, heroin, or club drugs: such as 3,4-methylenedioxymethamphetamine, ecstasy, gamma-hydroxybutyrate, Rohypnol, ketamine and roofies, stimulants, or “other drugs”).

### Bias

We chose 12-month (and not lifetime) variants when applicable to minimize recall bias.

### Statistical Analysis

Statistical analysis was performed in “R” version 3.6.1 using the “survey” package (25, 26) to implement the analysis of NESARC-III complex survey design (Taylor series linearization).

Descriptive statistics were computed for 12-month cannabis users in two population subgroups. Those include population share of sociodemographic characteristics and prevalence of psychiatric disorders, SUDs, and medical conditions. Proportion comparison hypothesis testing was done using χ^2^ test for survey data (“svychisq” function), and mean comparison hypothesis testing was done using survey data *t*-test (“svyttest” function).

We present predictive marginal means for the prevalence of past-year CUD and CUD severity levels, use characteristics, and prevalence of CUD criteria. For each variable, depending on its type, logistic regression or linear regression was estimated, with the characteristics as the dependent variable, while controlling for sex, race/ ethnicity, age, marital status, educational level, household income, urbanicity, and region. Marginal means were calculated (“svypredmeans” function) using a generalized linear model (“svyglm” function) with “Poisson” distribution with an “identity” link function or “quasibinomial” distribution with a “logistic” link function for factor variables. Marginal means for the two groups were compared using a *t*-test.

Linear regressions were used to measure the impact of different characteristics on the number of DSM-5 CUD criteria and thus on the chances of meeting criteria for CUD. For example, suppose the coefficient for characteristic X equals 1, a participant with X will have one criterion more than an equivalent participant without characteristic X. We present the association between the number of DSM-5 CUD criteria and respondents' sociodemographic characteristics, dosage, mental disorders, other SUDs, and medical conditions, for both RUs and medical users who also reported RU. Linear regressions were estimated using a generalized linear model with a “Poisson” distribution with an “identity” link function (“svyglm” function). All regressions (except the sociodemographic characteristic regression) are controlled for sociodemographic characteristics. For the dosage, mental disorders, other SUDs, and medical conditions analysis, *p*-values are adjusted using the Bonferroni correction. Regression coefficients were transformed into standardized effect sizes equivalent to Cohen's d by dividing the coefficient by the standard deviation of the model residuals.

All results were considered statistically significant if the *p*-value was below 0.05.

## Results

### Sample Characteristics

In our sample, 3,701 respondents reported past-year recreational cannabis use. Of them, 3,339 (91.1%) respondents reported RU only, and 362 (8.9%) reported RMU. The majority of users in both groups were men, the mean age of RU was 33.8 (SE = 0.370) and of RMU was 39.9 (SE = 0.977). Both groups were statistically similar in the majority of the sociodemographic variables. However, RMU tended to be older and more educated. Another statistically significant difference was in marital status. The RMU group has a higher percentage of married/in common law and people who are divorced, widowed, or separated. Also, the RU group has a higher rate of people who were never married. Psychiatric disorders and medical conditions were more common among RMUs. The two groups had no significant difference in the prevalence of SUDs. Sample characteristics are presented in Table 1.

**Table 1:** Characteristics of 12-month cannabis users in two population subgroups.

**Variable name**	**Recreational users only** ***N*** **=** **3,339**		**Recreational and** **medical users** ***N*** **=** **362**		**Mean** **difference** **test**
	***N***	**% (SD)**	***N***	**% (SD)**	***p*** **-value**
**Sociodemographic characteristics**					
**Sex**					
Female	1,384	37.9 (4.9)	136	36.3 (4.8)	0.627
Male	1,955	62.1 (4.9)	226	63.7 (4.8)	0.627
**Race**					
White	1,726	65.7 (4.7)	196	62.5 (4.8)	0.316
Other	1,613	34.3 (4.7)	166	37.5 (4.8)	0.316
**Marital status**					
Married/common law	885	32.4 (4.7)	124	41.6 (4.9)	0.007^**^
Widowed/divorced/separated	674	16.5 (3.7)	86	22.8 (4.2)	0.027^*^
Never married	1,780	51.1 (5.0)	152	35.6 (4.8)	<0.001^***^
**Age, years**					
18–29	1,519	49.7 (5.0)	119	33.8 (4.7)	<0.001^***^
30–44	983	27.1 (4.4)	117	30.4 (4.6)	0.219
45–90	837	23.2 (4.2)	126	35.8 (4.8)	0.003^**^
**Educational level**					
Some high school or less	517	13.6 (3.4)	40	9.9 (3.0)	0.056
High school graduate	941	28.6 (4.5)	89	24.4 (4.3)	0.098
Some college or higher	1,881	57.8 (4.9)	233	65.7 (4.8)	0.009^**^
**Household income**					
$0–19,999	1,253	31.7 (4.7)	139	36.4 (4.8)	0.232
$20,000–34,999	704	19.0 (3.9)	78	18.1 (3.9)	0.750
$35,000–69,999	825	25.7 (4.4)	88	25.2 (4.3)	0.839
$70,000 or greater	557	23.5 (4.2)	57	20.3 (4.0)	0.338
**Urbanicity**					
Urban	2,932	84.0 (3.7)	314	82.5 (3.8)	0.716
Rural	407	16.0 (3.7)	48	17.5 (3.8)	0.716
**Psychiatric disorders** ^a^					
Anxiety disorder	512	16.1 (3.7)	82	26.8 (4.4)	0.002^**^
Posttraumatic stress disorder	337	10.0 (3.0)	54	15.9 (3.7)	0.033^*^
Any mood disorder	796	24.1 (4.3)	103	28.3 (4.5)	0.117
Psychotic disorder	55	1.7 (1.3)	11	3.4 (1.8)	0.177
Personality disorder	966	30.0 (4.6)	147	42.1 (4.9)	0.001^**^
**Substance use disorde** ^b^					
Prior cannabis use disorder	658	20.8 (4.1)	87	25.4 (4.4)	0.141
Any other substance use disorder	301	9.4 (2.9)	46	12.3 (3.3)	0.181
Alcohol use disorder	1,537	47.9 (5.0)	158	42.6 (5.0)	0.128
Tobacco use disorder	1,617	49.4 (5.0)	180	50.6 (5.0)	0.744
**Medical conditions** ^c^					
Pain	713	19.7 (4.0)	126	35.8 (4.8)	<0.001^***^
Arthritis	340	9.2 (2.9)	79	23.4 (4.2)	0.001^**^
Nerve	322	9.7 (3.0)	71	21.1 (4.1)	<0.001^***^
Insomnia	247	6.9 (2.5)	55	17.5 (3.8)	0.001^**^
Cancer	63	1.7 (1.3)	18	5.0 (2.2)	0.006^**^
Diabetes	107	2.7 (1.6)	19	5.5 (2.3)	0.132

*** *p < 0.001*.

** *p < 0.01*.

* *p < 0.05*.

a *All psychiatric disorders refer to past-year, besides personality disorder that refers to lifetime diagnosis*.

b *All substance use disorders refer to past-year, besides prior cannabis use disorder that refers to before last year's diagnosis*.

c *All medical conditions refer to past-year, besides pain that refers to last 4 weeks. Past-year mood disorders include major depressive disorder, dysthymia, and bipolar 1. Past-year anxiety disorders include generalized anxiety disorder, social phobia, agoraphobia, specific phobias, and panic disorder. Lifetime personality disorders include borderline, schizotypal, and antisocial personality disorder. “Nerve” problems include fibromyalgia, reflex sympathetic dystrophy or complex regional pain syndrome, and any other nerve problem in your legs, arms, or back*.

### Medical Cannabis Use Disorder Severity and Characteristics of Cannabis Use

Past year CUD in total and in the three severities were more common among RMUs. The differences reached statistical significance for CUD total, mild and moderate CUD, but not for severe CUD category. The results partially support H1, which anticipated a higher rate of severe CUD among RMUs compared with RUs. Our results show that 46.8% (SE = 0.36) of RMUs met the criteria for CUD and 24.9% (SE = 0.10) from RUs. Among RUs and RMUs, the 12-month prevalence values of mild CUD are 13.5% (SE = 0.07) and 26.2% (SE = 0.35), the prevalence values of moderate CUD are 5.7% (SE = 0.05) and 12.9% (SE = 0.24), and the prevalence values of severe CUD are 5.7% (SE = 0.05) and 8.9% (SE = 0.21), respectively.

The two groups are also significantly different in the frequency and amount of use. After controlling for sociodemographic variables, almost 60% of RMUs endorsed daily use, whereas only around a quarter of RUs endorsed daily use. Also, on average, an RMU will use 1.1 joints per day, more than the average quantity reported for RU.

The results are presented in Table 2.

**Table 2:** Past-year cannabis use disorder (CUD) prevalence, by severity level, and cannabis use characteristics among past-year cannabis users in two population subgroups.

**Variable name**	**Recreational users only**	**Medical and recreational users**	**Mean difference test**
	**% (SE)**	**% (SE)**	***p*** **-value**
**CUD severity level**			
Cud mild	13.5 (0.07)	26.2 (0.35)	<0.001^***^
CUD moderate	5.7 (0.05)	12.9 (0.24)	0.004^**^
CUD severe	5.7 (0.05)	8.9 (0.21)	0.150
CUD total	24.9 (0.10)	46.8 (0.36)	<0.001^***^
**Use characteristics**			
Daily use	26.4 (0.10)	59.2 (0.29)	<0.001^***^
Numbers of joints per day	1.9 (0.05)	3.0 (0.20)	<0.001^***^
**Past-year cannabis use disorder criteria**			
Larger amounts/longer period than intended	5.3 (0.05)	7.0 (0.16)	0.311
Unsuccessful tries/wish to stop/cut down	17.7 (0.08)	21.9 (0.26)	0.137
Spent a great deal of time	10.7 (0.08)	23.3 (0.41)	0.003^**^
Strong urge/desire	17.1 (0.08)	34.0 (0.37)	<0.001^***^
Problems fulfilling your role	2.4 (0.03)	4.5 (0.17)	0.212
Social/interpersonal problems	7.0 (0.06)	14.3 (0.23)	0.002^**^
Activities given up	3.6 (0.04)	6.7 (0.20)	0.126
Hazardous use	23.4 (0.10)	38.3 (0.35)	<0.001^***^
Physical/psychological problems	8.1 (0.05)	10.4 (0.24)	0.354
Tolerance	14.2 (0.08)	30.1 (0.31)	<0.001^***^
Withdrawal	9.0 (0.07)	13.6 (0.21)	0.041^*^

*** *p < 0.001*.

** *p < 0.01*.

* *p < 0.05*.

*For each row, depending on type of variable, logistic regression or linear regression was estimated, with characteristics as dependent variable, while controlling for sex, race/ethnicity, age, marital status, educational level, household income, urbanicity, and region. Values presented are estimated marginal means and their standard errors (SE), and p-values are from t-test that accounts for survey design and weights*.

### Prevalence of Different Diagnostic and Statistical Manual of Mental Disorders, Fifth Edition, Cannabis Use Disorder Criteria

All the criteria were more prevalent among RMUs. However, only the following CUD DSM-5(19) criteria reach statistical significance: spending a great deal of time on activities related to cannabis, strong urge or desire to use cannabis, using cannabis despite social/interpersonal problems related to cannabis use, hazardous use, having tolerance to the effect of cannabis, and withdrawal symptoms when not using cannabis. The results support our H2 and are presented in Table 2.

### Association Between Respondents' Sociodemographic Characteristics on the Number of Cannabis Use Disorder Criteria

Results are shown in Table 3. On average, young, male, and racial/ethnic minorities met more CUD criteria in both the RU and RMU groups. However, the magnitude was higher among RMUs. For example, on average, men from the RMU group met 0.37 CUD criterion (Cohen's d = 0.26) more than women from that group, whereas, in the RU group, male use had 0.18 criterion (Cohen's d = 0.12) more than a female user. RMU living in the Midwest had a greater risk for CUD, whereas RU residing in the south had a greater risk. RUs with an annual household income of < $20,000 and high school graduates had a higher risk for CUD. The findings partially support H3.

**Table 3:** Association between respondents' sociodemographic characteristics and number of past-year cannabis use disorder criteria (Diagnostic and Statistical Manual of Mental Disorders, Fifth Edition).

**Variable**	**Recreational users only**				**Recreational and medical users**			
	**Coef**.	**Cohen's d**	***p*** **-value**	**CI 95%**	**Coef**.	**Cohen's d**	***p*** **-value**	**CI 95%**
**Sex**								
Male	0.18^*^	0.12	0.010	(0.04, 0.32)	0.37^**^	0.26	0.002	(0.15, 0.58)
Female	Reference							
**Race/ethnicity**								
White	−0.30^***^	−0.19	<0.001	(−0.44, −0.15)	−0.38^*^	−0.27	0.014	(−0.68, −0.09)
Other	Reference							
**Age, years**								
18–29	0.55^***^	0.36	<0.001	(0.29, 0.82)	0.91^**^	0.65	0.002	(0.40, 1.43)
30–44	0.31^*^	0.21	0.017	(0.06, 0.57)	0.67^*^	0.48	0.020	(0.12, 1.23)
45–90	Reference							
**Marital status**								
Widowed/divorced/ separated	0.14	0.09	0.284	(−0.12, 0.41)	−0.31	−0.22	0.322	(−0.95, 0.33)
Never married	0.10	0.06	0.180	(−0.05, 0.24)	0.00	0.00	0.992	(−0.37, 0.37)
Married/ common law	Reference							
**Educational level**								
Some high school or less	0.18	0.12	0.076	(−0.02, 0.39)	−0.09	−0.06	0.584	(−0.41, 0.24)
High school graduate	0.16^*^	0.10	0.024	(0.02, 0.29)	−0.04	−0.03	0.821	(−0.36, 0.29)
Some college or higher	Reference							
**Household income**								
$0–19,999	0.27^*^	0.17	0.018	(0.05, 0.49)	0.26	0.18	0.309	(−0.26, 0.78)
$20,000–34,999	0.17	0.11	0.150	(−0.06, 0.40)	−0.13	−0.09	0.680	(−0.76, 0.51)
$35,000–69,999	0.04	0.03	0.709	(−0.18, 0.27)	0.00	0.00	0.987	(−0.61, 0.60)
$70,000 or greater	Reference							
**Urbanicity**								
Urban	−0.16	−0.10	0.134	(−0.36, 0.05)	0.27	0.19	0.135	(−0.09, 0.63)
Rural	Reference							
**Region**								
Northeast	0.17	0.11	0.152	(−0.06, 0.39)	0.30	0.21	0.156	(−0.13, 0.73)
Midwest	0.11	0.07	0.354	(−0.13, 0.35)	0.48^**^	0.34	0.005	(0.16, 0.79)
South	0.21^*^	0.14	0.033	(0.02, 0.40)	0.03	0.02	0.888	(−0.37, 0.42)
West	Reference							
*N*	3,339				362			

*** *p < 0.001*.

** *p < 0.01*.

* *p < 0.05*.

*Co-efficient measures marginal addition to number of DSM-5 CUD criteria. Regression coefficients were transformed into standardized effect sizes equivalent to Cohen's d by dividing coefficient by standard deviation of model residuals*.

### Impact of Respondents' Comorbidity and Frequency and Amount of Use on Number of Cannabis Use Disorder Criteria

Although the amount of use was significantly associated with more CUD criteria in both groups, use frequency was associated with an increased likelihood to meet CUD criteria in both groups but reached statistical significance only among RUs and not among RMUs. Results are shown in Table 4A.

**Table 4:** Association between respondents' mental, medical, and substance use morbidity, frequency and amount of use, and number of cannabis use disorder criteria (Diagnostic and Statistical Manual of Mental Disorders, Fifth Edition).

**Variable**	**Recreational users only**				**Recreational and medical users**			
	**Coef**.	**Cohen's d**	***p*** **-value**	**CI 95%**	**Coef**.	**Cohen's d**	***p*** **-value**	**CI 95%**
**A. Frequency and amount of cannabis use**								
Amount used	0.10^***^	0.06	<0.001	(0.08, 0.11)	0.08^**^	0.06	0.001	(0.04, 0.12)
Frequency of use	1.02^***^	0.70	<0.001	(0.88, 1.15)	0.32	0.23	0.173	(−0.05, 0.70)
**B. Psychiatric disorders** ^a^								
Anxiety disorder	0.53^***^	0.35	<0.001	(0.36, 0.70)	0.33	0.24	0.431	(−0.05, 0.72)
Posttraumatic stress disorder	0.70^***^	0.46	<0.001	(0.53, 0.87)	0.76^*^	0.57	0.013	(0.31, 1.21)
Any mood disorder	0.75^***^	0.50	<0.001	(0.60, 0.89)	0.66^**^	0.49	0.005	(0.32, 1.01)
Psychotic disorder	1.01^***^	0.66	<0.001	(0.67, 1.35)	0.89^**^	0.65	0.003	(0.45, 1.32)
Personality disorder	0.61^***^	0.40	<0.001	(0.46, 0.76)	0.51^*^	0.37	0.027	(0.18, 0.85)
**C. Substance use disorder** ^b^								
Prior cannabis use disorder	1.39^***^	1.03	<0.001	(1.27, 1.52)	1.08^***^	0.87	<0.001	(0.83, 1.33)
Any other substance use disorder	0.63^***^	0.41	<0.001	(0.42, 0.84)	0.26	0.18	0.527	(−0.09, 0.60)
Alcohol use disorder	0.51^***^	0.34	<0.001	(0.36, 0.65)	0.22	0.16	0.531	(−0.07, 0.51)
Tobacco use disorder	0.67^***^	0.45	<0.001	(0.52, 0.83)	0.39	0.28	0.146	(0.03, 0.75)
**D. Medical conditions** ^c^								
Pain	0.23	0.15	0.088	(0.05, 0.42)	−0.07	−0.05	1.000	(−0.47, 0.32)
Arthritis	−0.14	−0.09	1.000	(−0.44, 0.16)	0.14	0.10	1.000	(−0.38, 0.65)
Nerve	0.10	0.07	1.000	(−0.15, 0.36)	0.08	0.05	1.000	(−0.43, 0.58)
Insomnia	0.11	0.07	1.000	(−0.16, 0.39)	0.33	0.24	1.000	(−0.19, 0.85)
Cancer	0.36	0.23	0.846	(−0.10, 0.81)	0.33	0.23	1.000	(−0.15, 0.81)
Diabetes	−0.07	−0.05	1.000	(−0.47, 0.33)	0.47	0.34	0.889	(−0.15, 1.09)

*** *p < 0.001*.

** *p < 0.01*.

* *p < 0.05*.

*Results are adjusted for sociodemographic variables. P-values are adjusted using Bonferroni correction for each subsection of table. Coefficient measures marginal addition to number of DSM-5 CUD criteria. Regression coefficients were transformed into standardized effect sizes equivalent to Cohen's d by dividing coefficient by standard deviation of model residuals*.

a *All psychiatric disorders refer to past-year, besides personality disorder that refers to lifetime diagnosis*.

b *All substance use disorders refer to past-year, besides prior cannabis use disorder that refers to before last year's diagnosis*.

c *All medical conditions refer to past-year, besides pain that refers to last 4 weeks. Past-year mood disorders include major depressive disorder, dysthymia, and bipolar 1. Past-year anxiety disorders include generalized anxiety disorder, social phobia, agoraphobia, specific phobias, and panic disorder. Lifetime personality disorders include borderline, schizotypal, and antisocial personality disorder. “Nerve” problems include fibromyalgia, reflex sympathetic dystrophy or complex regional pain syndrome, and any other nerve problem in your legs, arms, or back*.

In both groups, psychiatric disorders were significantly associated with more CUD criteria. The only variable that did not reach statistical significance was past-year any anxiety disorder, among RMUs. Results are shown in Table 4B.

All SUDs in question were significantly associated with meeting more CUD criteria among RUs. Among RMUs, all of the SUD's checked were associated with an increased likelihood of meeting more CUD criteria; however, only before the past-year CUD reached statistical significance. Results are shown in Table 4C.

Interestingly, none of the medical conditions examined in both groups reached statistical significance to meet more or less CUD criteria. The findings do not support H3 and are shown in Table 4D.

## Discussion

In light of the ongoing changes in MC's legislation, when more jurisdictions allow the use of cannabis for medical purposes (1, 2), we conducted a study using nationally representative epidemiological data to investigate the characteristics of CUD among RMUs and RUs. We assessed the prevalence of DSM-5 CUD in general and in all severities (mild, moderate, and severe). The prevalence of the different DSM-5 CUD criteria and the different personal characteristics (sociodemographic variables, the amount and frequency of cannabis use, mental and SUDs, and medical conditions) are associated with CUD.

Our results show that past-year CUD, in general, and in all severity categories, is more common among RMUs compared with RUs. The differences reached statistical significance for CUD total, mild and moderate CUD, but not for severe CUD category. This result might imply a lack of power, as only 26 RMUs met the criteria for severe CUD. Of note, the 2013 NSDUH, another American national survey, found that the differences in CUD between medical and RUs are smaller (11% among medical users and 10% among RUs) and that the difference does not reach statistical significance (5). This inconsistency can be related to several differences. First, the NSDUH used DSM-IV criteria assessing cannabis problematic use. Second, the daily use rate in our study was much higher in both groups in question, thus might lead to a higher risk for dependency. Third, our study focused on recreational cannabis users and excluded participants who used cannabis only medically, whereas the study based on NSDUH data also included participants who used cannabis only medically.

Compared with RUs, RMUs consumed more cannabis: they used cannabis more frequently and used a greater amount while using. This is consistent with prior literature (5, 6). The increased cannabis consumption can result from increased accessibility to cannabis among medical users and increase the risk of cannabis-related harms, such as CUD. Future studies should investigate the impact of changes in cannabis legislation on people who use cannabis for medical purposes and if people with medical conditions are affected differently than people without medical conditions.

So far, there is no literature examining the validity of CUD diagnostic criteria among MC users. One might argue that pharmacological dependency (i.e., tolerance or withdrawal) should be expected in cases of long-term regular use of medication. For example, that was the consensus among pain doctors in a similar situation, people who use prescribed opioids (27). On the contrary, a recent critical review on this subject stated that we should see tolerance, significant withdrawal symptoms, and craving to prove dependence in medical users (14). In our study, all participants also endorsed recreational cannabis use. Thus, the lack of validity for CUD among medical users only partially applies to this group. However, cautiousness is still warranted.

In our data, all the criteria for CUD were more prevalent among RMUs compared with RUs. For 6 of the 11, the difference was statistically significant. Around 30% of RMUs developed tolerance to cannabis, and 13.6% reported having withdrawal symptoms related to cannabis. As mentioned, both criteria were more prevalent among RMUs compared with RUs.

There are also several other findings worth attention. The most prevalent criterion among RMU is hazardous use; 38.3% of RMUs endorsed it (compared with 23.4% of RUs). Chronic cannabis users perform worse on driving than non-users (28). Thus, this result might pose a significant public safety risk. In addition, 34% of RMUs, compared with 17.1% of the RUs, reported strong urge or desire to use cannabis; 14.3% of the RMUs endorsed social and interpersonal problems related to cannabis use, compared with only 7% of RUs; 23.3% of RMUs, compared with 10.7% of RUs, reported spending a great deal of time on activities related to cannabis. Future studies should continue to develop the symptoms likely to capture dependence in the medicinal context. It is also important to assess the benefit of MC use. For example, evidence suggests that MC can diminish prescription opioid usage (29). Thus, cannabis may be a safer alternative for managing chronic pain.

We also analyzed which personal characteristics are associated with CUD among the RMUs and RUs. Overall, most of the sociodemographic characteristics of the impact CUD risk overlapped between populations. Being a male, young age, and not white were individually correlated with increased risk in both groups, but the magnitude was higher among RMUs. Future research should assess the differences in magnitude and whether there are psychological factors associated with RMUs that may increase the risk for CUD.

Although the amount of cannabis used was linked with more CUD criteria in both groups, the frequency of use reached significance only in the RU group. This difference probably stems from the fact that frequent use is common among medical users (5, 6) and therefore does not differentiate between users with and without CUD.

In both groups, psychiatric disorders were significantly associated with more CUD criteria. The only variable that did not reach statistical significance was past-year any anxiety disorder, among RMUs. Those results are in line with previous literature that reported high comorbidity between CUD and mental disorders (30, 31).

Interestingly, our study showed that only before past-year CUD was significantly associated with more CUD criteria, whereas all the SUDs in questions were associated with more CUD criteria among RUs. This finding should also be explored in future research.

Our results did not find any significant impact of physical morbidity on the risk for CUD in both populations. This result should also be taken cautiously, given that the data related to medical conditions lack the severity of the disease or the duration of morbidity.

Several additional limitations of the study should be highlighted. First, the data in our study are cross-sectional, and causality cannot be inferred. Second, data in NESARC-III are based on self-reports; thus, misreporting might be a concern. However, previous studies have pointed out that anonymous surveys have a low misreporting share (32). Third, in the sample that we used, 79.15% of the medical users also reported RU (11). We focused on this group, and our results do not apply to individuals who use cannabis exclusively for medical purposes. Fourth, the survey was done in the United States and did not necessarily represent other populations or other countries. Fifth, NESARC-III does not include data related to cannabis potency and way of administration. Finally, NESARC-III data were collected in 2012–2013. Given the rapid global changes in medical and recreational cannabis use, it might not reflect current cannabis use patterns, such as use-modes and cannabis potency.

Despite those limitations, our study provides valuable insights into the prevalence and characteristics of CUD among RMUs. With an increasing number of RMUs, this study represents an important first step in characterizing CUD among people who use MC. It is highly important that health care workers who prescribe or recommend the use of cannabis for medical purposes will thoroughly consider the ratio between the potential risk and benefit, assess RU, and educate and screen patients for CUD and other complications that may be caused by cannabis use. We hope that future studies will shed more light on the validity of CUD diagnostic criteria in the context of long-term MC use. Future studies should also assess data related to CUD among exclusive medical users and continue to explore the psychological and cognitive factors linked with increased risk for CUD. In addition, conducting longitudinal studies, studies outside of the United States, and studies with more medical users will help to build a body of literature on the complications and risks faced by MC users.

In summary, this study suggests that past-year CUD is more prevalent among RMUs, in general, as well as mild and moderate CUD compared with RUs. CUD is more prevalent in RMUs, with the following characteristics: young, male, non-white, living in the Midwest, using a greater amount of cannabis, had a mood/psychotic disorder/post-traumatic stress disorder in the past year, have a personality disorder, or had CUD before the past year.

## Data Availability Statement

Publicly available datasets were analyzed in this study. This data can be found here: https://www.niaaa.nih.gov/research/nesarc-iii.

## Ethics Statement

NESARC-III was approved by the Institutional Review Boards at the National Institutes of Health. The study presented in this manuscript received approval from the Centre for Addiction and Mental Health's Research Ethics Board (099-2019-01). Written informed consent for participation was not required for this study in accordance with the national legislation and the institutional requirements.

## Author Contributions

DR-K, AH, and BL contributed to the conceptualization and methodology. DR-K and MS contributed to the statistical analysis. DR-K contributed to writing the original draft preparation. All authors contributed to the review and editing, read, and approved the final manuscript.

## Conflict of Interest

BL has obtained funding from Pfizer Inc. (GRAND Awards, including salary support) for investigator-initiated projects. BL has obtained funding from Indivior for a clinical trial sponsored by Indivior. BL has in-kind donations of cannabis products from Aurora Cannabis Enterprises Inc. and study medication donations from Pfizer Inc. (varenicline for smoking cessation) and Bioprojet Pharma. He was also provided a coil for a Transcranial magnetic stimulation (TMS) study from Brainsway. BL has obtained industry funding from Canopy Growth Corporation (through research grants handled by the Centre for Addiction and Mental Health and the University of Toronto), Bioprojet Pharma, Alcohol Countermeasure Systems (ACS), and Alkermes. Lastly, BL has received in kind donations of nabiximols from GW Pharmaceuticals for past studies funded by CIHR and NIH. He has participated in a session of a National Advisory Board Meeting (Emerging Trends BUP-XR) for Indivior Canada and has been consultant for Shinogi. He is supported by CAMH, a clinician-scientist award from the department of Family and Community Medicine of the University of Toronto and a Chair in Addiction Psychiatry from the department of Psychiatry of University of Toronto. The remaining authors declare that the research was conducted in the absence of any commercial or financial relationships that could be construed as a potential conflict of interest.

## Publisher's Note

All claims expressed in this article are solely those of the authors and do not necessarily represent those of their affiliated organizations, or those of the publisher, the editors and the reviewers. Any product that may be evaluated in this article, or claim that may be made by its manufacturer, is not guaranteed or endorsed by the publisher.

## Footnotes

^1^Group quarters are places where individuals can live or stay in a group living arrangement. The group quarters are owned or managed by an organization/entity providing housing and services for the residents.

^2^We decided to divide the race/ ethnicity to White vs other, as other ethnic groups (Black, Native American, Asian/ Pacific Islander, Hispanic) had a small representation in the RMU group.

^3^In the hierarchical diagnosis, exclusionary criteria from DSM-5 were applied, thus substance-induced mood episode or an episode due to general medical condition were excluded.
